# Estimating hybridization in the presence of coalescence using phylogenetic intraspecific sampling

**DOI:** 10.1186/1471-2148-11-291

**Published:** 2011-10-06

**Authors:** David Gerard, H Lisle Gibbs, Laura Kubatko

**Affiliations:** 1 Department of Statistics, The Ohio State University, Columbus, OH 43210, USA; 2 Department of Evolution, Ecology, and Organismal Biology, The Ohio State University, Columbus OH 43210, USA

## Abstract

**Background:**

A well-known characteristic of multi-locus data is that each locus has its own phylogenetic history which may differ substantially from the overall phylogenetic history of the species. Although the possibility that this arises through incomplete lineage sorting is often incorporated in models for the species-level phylogeny, it is much less common for hybridization to also be formally included in such models.

**Results:**

We have modified the evolutionary model of Meng and Kubatko (2009) to incorporate intraspecific sampling of multiple individuals for estimation of speciation times and times of hybridization events for testing for hybridization in the presence of incomplete lineage sorting. We have also utilized a more efficient algorithm for obtaining our estimates. Using simulations, we demonstrate that our approach performs well under conditions motivated by an empirical data set for *Sistrurus *rattlesnakes where putative hybridization has occurred. We further demonstrate that the method is able to accurately detect the signature of hybridization in the data, while this signal may be obscured when other species-tree inference methods that ignore hybridization are used.

**Conclusions:**

Our approach is shown to be powerful in detecting hybridization when it is present. When applied to the *Sistrurus *data, we find no evidence of hybridization; instead, it appears that putative hybrid snakes in Missouri are most likely pure *S. catenatus tergeminus *in origin, which has significant conservation implications.

## Background

In multi-gene phylogenetic analyses, gene phylogenies, which represent the evolutionary histories of particular genes, commonly differ from the overall species phylogeny, which represents the evolutionary relationships of the organisms as a whole. Multiple biological phenomena have been proposed to explain such incongruence, including hybridization [1], deep coalescence (also called incomplete lineage sorting (ILS)), recombination [2], horizontal gene transfer [3], and gene extinction and duplication [4]. Clearly, the possibility that such processes contribute to observed incongruence in gene trees complicates the inference of species phylogenies from multilocus data [5]. Most of the currently used methods for species tree estimation focus on modeling only one source of incongruence. For example, several software packages have been developed to estimate species phylogenies under the assumption that ILS is the only process contributing to variability in the underlying gene trees [6-9].

However, others have attempted to consider more than one process simultaneously. In particular, several groups have considered both hybridization and ILS in a phylogenetic framework [10-15]. In this paper, we consider this problem in the case in which several individuals are sampled from each species of interest, and gene phylogenies without branch length information are used to assess the support for hybridization in the presence of ILS. We do this by extending the method of Meng and Kubatko [11] in two ways. First, we allow for the possibility of intraspecific sampling of any number of individuals within each species, and second we provide a more efficient algorithm for estimation of speciation times and times of hybridization events. The newly-developed methodology has been implemented in a perl script that is available for download from http://www.stat.osu.edu/~lkubatko/software/HybTree/.

Our work is motivated by empirical studies of North American Massasauga rattlesnakes (*Sistrurus catenatus*), a group for which putative hybrids have been identified. Massasauga rattlesnakes are currently classified into three distinct subspecies: *Sistrurus catenatus catenatus*, found in eastern North America east of the Mississippi River, *S. c. tergeminus*, found in the central United States (Kansas, Oklahoma, and Texas), and *S. c. edwardsii*, found in the southwestern United States (Arizona and New Mexico). A recent analysis of this species based on 18 nuclear genes and one mitochondrial gene found evidence for genetic distinctiveness of these three subspecies [16], with strongest support for distinctiveness of *S. c. catenatus*, the eastern Massasauga, from the two western subspecies. This finding is important because the eastern Massasauga is a candidate for listing as a Federally Threatened or Endangered Species in the United States.

In northwest and central Missouri, Gloyd [17] and Evans and Gloyd [18] identified populations containing individuals that were morphologically intermediate between two subspecies (*S. c. catenatus *and *S. c. tergeminus*) with adjacent distributions to the east and west, respectively, of these populations (see Gibbs et al. [19] for a complete description of the possible hybrid zone). Although populations in this area are currently classified as *S. c. catenatus *[20] it is unknown if these snakes are true genetic hybrids or whether their morphological intermediacy is due to other evolutionary or ecological factors. In addition to a possible hybrid origin of these populations, Gibbs et al. [19] provided genetic evidence based on microsatellite and mitochondrial markers that individuals in the Missouri populations may be pure *S. c. tergeminus *individuals. Here, we further examine the question of the hybrid origin of these populations by applying our newly-developed methodology to data collected from 12 of the genes previously used by Kubatko et al. [16].

We begin by first carrying out a simulation study to assess the ability of the newly proposed method to detect hybridization in the presence of ILS for genetic data that mimics that available in our empirical study. We then apply our methodology to the empirical data to address the question of hybridization in the ancestry of the Massasauga rattlesnakes from Missouri. In addition to testing our method, we analyze both our simulated and empirical data using one of the popular methods for species tree inference, BEST (Bayesian Estimation of Species Trees; [6]). BEST explicitly models ILS, but does not incorporate hybridization. We then apply both of these methods to the empirical data to address the question of hybridization in the ancestry of the Massasauga rattlesnakes from Missouri.

## Results

### Hybridization Model

In the context of the coalescent process [21,22], Meng and Kubatko [11] describe a model in which, given a species tree topology and a set of gene tree topologies assumed to be correct, estimates of the proportion of hybridization, *γ*, may be obtained for an a priori specified hybrid population. Their method additionally provides estimates of the branch lengths along the known species tree topology, and proposes a likelihood ratio test of the null hypothesis that the putative hybrid population is derived entirely from one parental species. Here, we present an approach that uses this same model but with two important modifications: first, it allows for the incorporation of multiple individuals per species; second, we replace the grid search for the maximum likelihood estimates of the branch lengths and the *γ *parameter used by Meng and Kubatko [11] with a search that utilizes Brent's method [23], an optimization technique for one-dimensional functions. For functions that are suitably parabolic near the extrema, Brent's method can find extrema rapidly [24]. We apply Brent's method iteratively until convergence criteria have been reached. Further detail about each of these steps is given in the Materials and Methods section.

### Simulation Study

**Figure 1:**
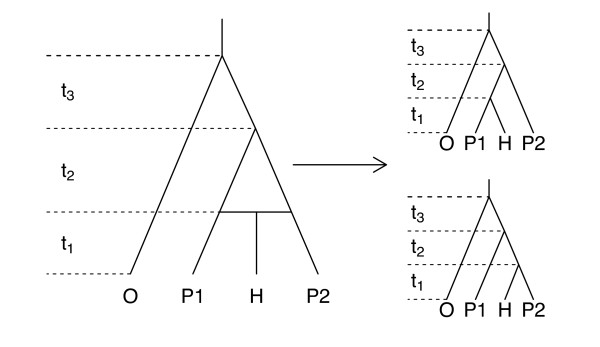
**Model Tree for the Simulation Study**. Model species tree for the simulation study. Taxon H is assumed to be a hybrid of taxa P1 and P2. The *t*_i _parameters give the interval of time (in coalescent units) between speciation events.

To examine the performance of our method of assessing evidence for hybridization in the presence of ILS, we first conducted a simulation study using the hybrid species phylogeny in Figure 1 as the model tree. Using the species tree in Figure 1, ten gene trees were simulated, with the number of gene trees derived from each parental species tree drawn from a Binomial distribution with probability equal to the true proportion of hybridization (*γ*). Sequence data were then simulated from each gene tree. Using this sequence data, the ten gene trees were estimated using maximum likelihood. These gene trees were used in our approach to estimate the proportion of hybridization of the putative hybrid species and the species tree branch lengths, and a likelihood ratio test was performed at level *α *= 0.05. We then applied BEST to the same simulated sequence data to calculate the posterior probability of the putative hybrid species sharing its most recent common ancestor with each parental taxon. We repeated this procedure 100 times each for four levels of hybridization (*γ *= 0.0, 0.1, 0.3, or 0.5). These simulation settings were selected in order to mimic the conditions under which our empirical data were obtained. In the Discussion, we comment on some limitations of these settings and speculate on how the method might be expected to perform in other situations.

**Figure 2:**
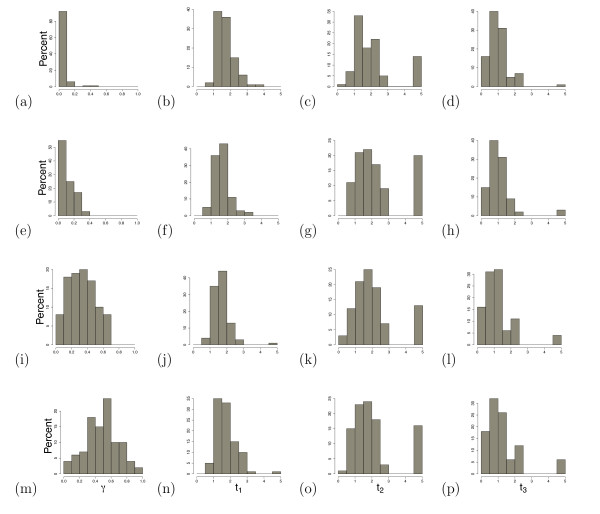
**Histograms of the MLEs in the Simulation Study**. Histograms of MLEs of parameters for the 100 trials in the simulation study. The rows each correspond to a specific value of *γ *(*γ *= 0, 0.1, 0.3, and 0.5, respectively). Columns correspond to the four parameters that are estimated in each trial (*γ*, *t*_1_, *t*_2_, and *t*_3_).

The results of this simulation study are shown in Table 1 and Figure 2. Specifically, the table gives the average values of the maximum likelihood estimates (MLEs) of the hybridization parameter *γ*, as well as the average MLEs of the branch lengths in the hybrid species tree, *t*_1_, *t*_2_, and *t*_3 _(averages are computed over the 100 replicate data sets generated for each value of *γ*), while Figure 2 shows histograms of these parameters. Standard deviations of the MLEs across the 100 replicates are also given. Overall, the average estimates of *γ *were close to their true values, indicating that the method has good ability to estimate the contribution from each parental taxon to the hybrid species. The standard deviations indicate a fair amount of variability in these estimates, but given that the simulation study was based on a sample of only 10 loci, this is not unexpected. The variability can be predicted to decrease as the number of loci sampled increases, as was observed in the simulation study of Meng and Kubatko [11] when only a single sequence per species was considered.

**Table 1:** Results of the Simulation Study

*γ*	**Mean **$\left(\hat{\gamma }\right)$	*SD *$\left(\hat{\gamma }\right)$	Mean $\left(\hat{t_{1}}\right)$	SD $\left(\hat{t_{1}}\right)$	Mean $\left(\hat{t_{2}}\right)$	SD $\left(\hat{t_{2}}\right)$	Mean $\left(\hat{t_{3}}\right)$	SD $\left(\hat{t_{3}}\right)$	Power
0	0.026	0.064	1.694	0.489	2.128	1.255	1.034	1.255	0.05
0.1	0.102	0.105	1.649	0.468	2.339	1.434	1.107	0.830	0.38
0.3	0.337	0.172	1.648	0.523	2.086	1.265	1.214	0.942	0.78
0.5	0.497	0.203	1.726	0.611	2.110	1.364	1.263	1.099	0.87

Results of the simulation study for varying *γ *using the model hybrid species tree in Figure 1. The value of *γ *used to simulate the data is shown in column 1. Columns 2 and 3 give the mean and standard deviation (SD) of the MLEs of *γ *over the 100 replicates. Similarly, columns 4 and 5, 6 and 7, and 8 and 9 give the means and SDs of the MLEs of the branch length estimates. Column 10 gives the power of the likelihood ratio test of the null hypothesis that *γ *= 0.

In contrast, the branch lengths were consistently overestimated by our method, especially near the node where hybridization occurred. This is at least in part explained by the fact that on some proportion of the the trials, the MLE of these branch lengths was estimated to be at the pre-defined boundary of 5.0 coalescent units (see Table 2 and Figure 2). However, examination of the distribution of the estimates in Figure 2 shows that even if these boundary cases are excluded, the MLEs of the branch lengths are upwardly biased. We return to this issue in the Discussion.

**Table 2:** Estimation of Branch Lengths at the Boundary

*γ*	***t***_**1**_	***t***_**2**_	***t***_**3**_
0.0	0	14	1
0.1	0	20	3
0.3	1	13	4
0.5	1	16	6

Information about the number of times branch lengths are estimated to be at the boundary in the simulated data set. Column 1 gives the value of *γ *used to simulate the data. Columns 2, 3, and 4 give the number of trials (of 100 total) for which the MLE of the particular branch length is estimated to be at the boundary.

The estimated power of the likelihood ratio test (the proportion of times the test rejects the null hypothesis of no hybridization) is shown in the far right column of Table 1. As expected, the power of the test increases as *γ *increases, indicating that it becomes easier to detect hybridization as the two parental species contribute more equally to the genome of the hybrid species. We also note that when there is no hybridization (*γ *= 0), the test achieves the desired 0.05 level.

**Figure 3:**
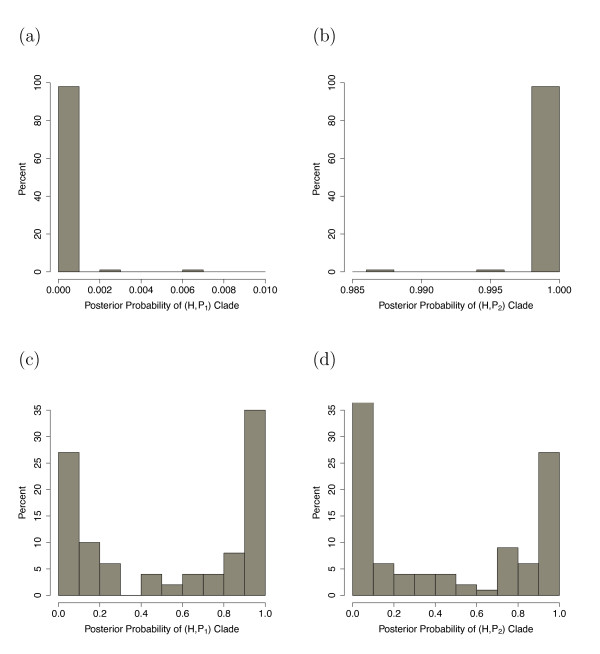
**Histograms of the Posterior Probabilities from BEST**. Histogram of posterior probabilities on nodes that are ancestral to the hybrid taxon in the 100 replicates of the simulation study. (a) and (b) give the results when the true value of *γ *is 0, and (c) and (d) give the results when *γ *is actually 0.5. (a) and (c) give the results for the clade containing taxa H and P1; (b) and (d) for the clade containing H and P2.

Table 3 shows the results of the application of BEST to the simulated data. We note that, on average, the posterior probabilities assigned to the nodes involved in the hybridization event reflect the true ancestry of the hybrid species. However, the variability in these probabilities is quite high, indicating that any one trial may not necessarily give an indication of the true history of hybridization. The histogram of posterior probabilities assigned to the nodes involved in the hybridization event (Figure 3) when *γ *= 0.5 is particularly interesting in that the posterior probability is often quite near 0 or 1, but that it is 0 or 1 approximately equally often, leading to the desired behavior on average. This result is not a statement of the "failure" of BEST by any means; rather it is an acknowledgment that using data generated from a model that is not included in BEST can lead to poor results in any particular trial, even though the average performance is reasonable and expected.

**Table 3:** Results of the Analysis with BEST

*γ*	APP (H,P1)	SDPP (H,P1)	APP (H,P2)	SDPP (H,P2)	APP (P1,P2)	SDPP (P1,P2)
0.0	0.000098	0.000691	0.9999	0.0014	0.000096	0.0007232
0.1	0.004096	0.01676	0.9940	0.0250	0.0019	0.0092
0.3	0.1799	0.2996	0.8129	0.3046	0.0073	0.0176
0.5	0.5289	0.4088	0.4647	0.4089	0.0064	0.0096

Results of the analysis of the simulated data using BEST [6]. Column 1 gives the value of *γ *used to simulate the data. Columns 2 and 3 give the average posterior probability (APP) and the standard deviation of the posterior probabilities (SDPP) assigned to the clade containing taxa *H *and *P*1 over the 100 trials. Columns 4 and 5, and 6 and 7, give the APP and SDPP for the clades *H *and *P*2, and *P*1 and *P*2, respectively.

### Empirical Data

**Figure 4:**
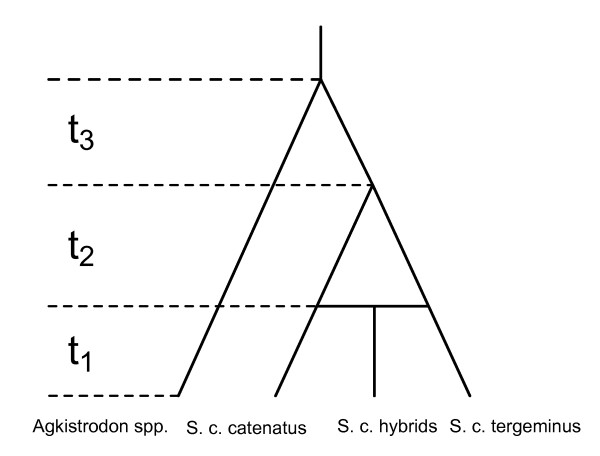
**Hybrid Species Tree for the Massasauga Rattlesnakes**. Hybrid species tree used to examine the evidence for hybridization between *S. c. catenatus *and *S. c. tergeminus *in the formation of putative hybrid populations of *Sistrurus *in Missouri.

To study the possibility of hybridization in the Missouri Massasauga populations, we used DNA sequence data for twelve of the genes analyzed by Kubatko et al. [16]. We included four individuals of pure *S. c. catenatus *and four of pure *S. c. tergeminus *orgin, as well as four individuals of putative hybrid origin. Two *Agkistrodon *individuals were used as the outgroup taxa. The fixed phylogeny relating these taxa and showing the putative hybridization event is given in Figure 4. Details of the loci and individuals used in the analysis are given in the Materials and Methods.

When we applied our method to this data set, we found that the MLE of *γ *was identically 0, indicating that the putative hybrid populations contained individuals that were entirely *S. c. tergeminus *in origin, with no shared ancestry with *S. c. catenatus*. Estimated branch lengths (in coalescent units) along the hybrid species phylogeny were *t*_1 _= 0.1114, *t*_2 _= 0.0945, and *t*_3 _= 2.0 (in this analysis, an upper bound of 2.0 was set on the coalescent times, so that the *t*_3 _parameter was estimated to be on the boundary). The rapid divergence times for *t*_1 _and *t*_2 _indicate that ILS is a much more likely explanation for any observed gene tree/species tree incongruence than hybridization. These results are in strong agreement with those found by Gibbs et al. [19], who showed using independent genetic data that putative hybrid individuals were most likely entirely *S. c. tergeminus *in origin.

Finally, we also analyzed the rattlesnake data set using BEST under the same settings used in the simulation study. The posterior probability of *S. c. tergeminus *and the potential hybrids as a clade was 0.988. The posterior probabilities of *S. c. catenatu***s **and the potential hybrids, and of *S. c. catenatus *and *S. c. tergeminus *each as distinct clades were low (0.009 and 0.003, respectively). These results are in agreement with our findings from using the model for hybridization.

## Discussion

Our results show a striking improvement in the power of the likelihood ratio test over that observed by Meng and Kubatko [11], suggesting that the incorporation of multiple samples per species has a significant impact on our ability to detect hybridization when it occurs. At a sample size of ten genes, they observed powers of 0.09, 0.19, 0.34, and 0.41 at true hybridization levels of 0.0, 0.1, 0.3, and 0.5, respectively, which are much lower than we observed (see our Table 1). Their study showed an increase in power when the number of genes increased from 10 to 50, and we might expect corresponding gains in power in our setting as well. However, it is not clear how the number of genes and the number of samples per taxon might interact, and this would need to be carefully examined in further simulation studies. In addition, the study of Meng and Kubatko [11] did not simulate variability in gene trees due to estimation from sequence data. They simulated gene trees directly, rather than simulating sequence data from gene trees and using the gene trees estimated from the sequence data as the data for estimating hybridization, as we have done here. Our results therefore indicate that even a small sample of genes can be used to effectively detect hybridization when intra-specific sampling within taxa is used. We hypothesize that sampling more individuals than examined in this study would increase the power of our method, though gains may diminish as the total sample size becomes large. Inclusion of any number of tips per species is allowed by the perl script posted online.

**Figure 5:**
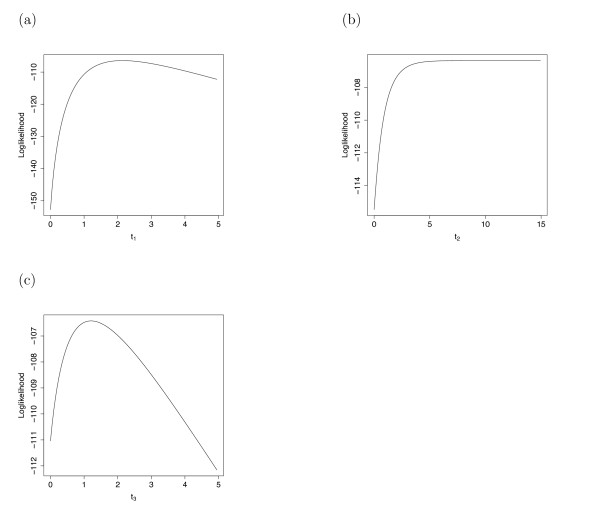
**The Likelihood Function for Each of the Model Parameters**. Plots of the likelihood function for each branch length with all other branch lengths held constant. Notice that the likelihood for *t*_2 _asymptotes and reaches the boundary condition.

While our method showed good ability to estimate *γ *and to test for evidence of hybridization in the presence of ILS, estimation of speciation times and the time of hybridization proved to be much more difficult. One problem we encountered was that speciation times were commonly estimated to be too large (often at the boundary that we set at 5.0 coalescent units). As an example, Figure 5 shows the likelihood as a function of each of the hybrid species tree branch lengths, as well as *γ*, holding the other branch lengths fixed, for one of the data sets simulated with *γ *= 0.5. We see that the likelihood as a function of *t*_2 _increases to an asymptotic level. In this data set, each gene tree topology is topologically concordant (in the sense of Rosenberg [25]) with one of the two species tree topologies. Hence, ILS alone is not needed to explain the observed gene tree/species tree incongruence, as all of the observed gene trees can be adequately explained by hybridization in the absence of ILS. The likelihood function is thus optimized as the probability of coalescence of all lineages approaches 1 along the branch with length *t*_2_, which occurs as *t*_2 _approaches infinity. At true branch lengths of 1.0 coalescent units, this type of data set occurs somewhat frequently under the coalescent model, particularly when the number of genes sampled is 10. We would expect that species tree branch lengths could be more accurately estimated in two situations. First, one could sample a larger number of genes, which would then provide increased information about branch lengths through the opportunity to observe more incongruence in gene trees. Second, shorter branch lengths in the species tree would lead to more observed incongruence among gene trees, thus again providing more information for branch length estimation.

However, even after excluding all data sets where boundary values are reached, the method still systematically overestimates branch lengths (see Figure 2). One possible explanation for this is our optimization technique, which involves the sequential optimization of one parameter at a time. In the four-dimensional domain of a complex likelihood function, it is possible that by first optimizing *γ *and then each branch length in turn, we are only approaching local, rather than global, maxima. While we could implement a simultaneous optimization procedure, we have not done this primarily because such an optimization scheme cannot easily be generalized to larger problems. This is consistent with the fact that most phylogenetic algorithms for optimization of branch lengths in single gene phylogenies (e.g., those implemented in programs like PAUP* [26] and PHYLIP [27]) find ML branch lengths by sequentially considering the branches. However, it is also possible that the optimization technique is not the issue - it may be that information from 10 gene tree topologies only (recall that gene tree branch lengths are not being used) is not sufficient to precisely estimate species tree branch lengths. Fan and Kubatko [28] also observed biases in the estimation of branch lengths when using only gene trees without branch length information in the inference procedure.

While the method proposed here does not use information concerning the gene tree branch lengths, methods that do incorporate gene tree branch length information to estimate times to speciation events have been developed (e.g., STEM [9]), and this information has been used to study hybridization as well [12]. In general, if accurate gene tree branch length information is available, these methods would be expected to perform better than the method proposed here, because the branch lengths would provide additional information about the species-level relationships. However, misleading information concerning gene tree branch lengths could cause bias in these methods, while leaving the method we propose here unaffected. Therefore, if the accuracy of branch length estimates is unknown, it would be better to use a simpler method which does not incorporate gene tree branch lengths. In particular, in cases where there has been recent, rapid divergence (often the case in situations where hybridization might be suspected), gene tree branch lengths are difficult to estimate accurately, and the method proposed here provides a reasonable alternative.

Our simulation settings were designed to mimic the properties of the empirical Massasauga data set, and thus are limited in some ways. For example, only hybridization between sister taxa was considered, and branch lengths in the model species tree were set to 1.0 coalescent units. In regard to hybridization between non-sister taxa, we might expect that this will be easier to distinguish from incomplete lineage sorting than a hybridization event between sister taxa would be, because mis-sorting of ancestral lineages is more common between closely related species. Indeed, this is what was found in the simulation study of Meng and Kubatko [11] when only a single individual per species was sampled. We also note that as branch lengths in the hybrid species tree increase, the amount of deep coalescence decreases since it becomes more probable that lineages coalesce in their ancestral populations. Thus it is expected that the longer the species tree branch lengths, the easier it will be to distinguish hybridization from incomplete lineage sorting. This is again supported by the results of Meng and Kubatko [11] for the case of a single individual.

Our approach could be extended in a number of ways. If multiple hybridization events are suspected to have occurred, one could incorporate a separate *γ *parameter for each putative hybridization event. The likelihood function is easily expanded to cover these situations (see [29] for an example). The extension is especially straightforward when hybridization events occur between distinct pairs of sister taxa. Complexities in calculations occur if multiple hybridization events occur along one branch in the tree, or in cases when hybridization events result from clades which have undergone hybridization events. Nakhleh et al. [15] have considered these kinds of situations in a parsimony framework.

When a putative hybrid taxon cannot be identified a priori, one could gain a greater understanding of gene flow between three species by iteratively using this method when each species is considered the putative hybrid. In the general case of *n *ingroup species and under the setting of one potential hybridization event, this would increase computational time by a factor of $3\times \left(\begin{array}{c} n\\ 3 \end{array}\right)$. This method is implemented in the script called ITERATE provided at the web address given above. Alternatively, given a known topology for a species tree one could iteratively perform this method on each (external) branch of the species tree to try to detect possible hybridization events across closely related species. If each tip was in turn considered the hybrid taxon and was placed at each possible internal branch as a hybrid arising from a relationship with the sister lineage of the selected internal branch, then $n\left(\frac{n-2}{2}\right)$ possible relationships would need to be considered.

We found that use of the posterior probability estimated by BEST provided a good indication of the extent of historical hybridization on average, but that the variability was large, indicating that the method may not be very informative on a case-by-case basis. While BEST is only one of a collection of possible methods for estimating species trees using multilocus data, we expect that other methods will perform similarly. In particular, *BEAST [7] also provides estimates of the posterior probability associated with clades in a coalescent-only framework, though using a different algorithm to carry out the Markov chain Monte Carlo, and thus we expect its performance to be similar. These results highlight the need for the development of methods that explicitly incorporate hybridization into the model, such as the one we propose here.

Our study provides genetic evidence that the hybrid populations of Massasauga rattlesnakes in Missouri are not hybrids at all, but rather entirely *S. c. tergeminus *in origin. Although our method may not, in general, be able to discriminate between hybridization and low levels of introgression, our results are confirmed by another analysis of an independent data set [19] that also came to the conclusion that the snakes in Missouri are not hybrids and that their morphological variation does not coincide with patterns of genetic differentiation. As noted by Gibbs et al. [19], this finding has several implications for the conservation status of these snakes. First, it indicates that *S. c. catenatus*, which is being considered for listing as a Federally Threatened or Endangered Species in the United States, has a lower range-wide total population size than previously believed since Missouri populations can no longer be included in this estimate. Second, it provides additional evidence against the classification of Massasauga populations in Missouri as the federally-listed *S. c. catenatus *since multiple sources of genetic data indicate that they are in fact *S. c. tergeminus*. However, state protection may be warranted as *S. c. tergeminus *is rare within Missouri [19].

## Conclusions

We have presented a new method for testing for hybridization or for estimating the extent of hybridization using multi-locus data with intraspecific samples among species. Using simulation, we have demonstrated that our method has good ability to detect hybridization when it is present, with increases in power due to increasing equality of contributions from both parental taxa and due to sampling more lineages within each species. While our method tends to overestimate times of speciation and hybridization, we expect estimates to improve as more genes are sampled. Our simulation studies also show that methods that do not explicitly model hybridization, such as BEST [6], may not give an accurate estimates of species relationships, thus highlighting the need for development of methods, such as the one presented here, that explicitly model hybridization in the presence of ILS.

When applied to an empirical data set for a hypothesized hybrid population of *Sistrurus *rattlesnakes, we fail to reject the hypothesis that ILS alone adequately explains the observed incongruence in single gene phylogenies. The estimate of the hybridization parameter indicates that the hybrid population is entirely *S. c. tergeminus *in origin, in agreement with the conclusions reached by Gibbs et al. [19] using different data and methodology. These findings have implications in Missouri and elsewhere for the conservation of Massasauga rattlesnakes.

## Methods

### Hybridization Model

Here we briefly discuss the model for hybridization in the presence of ILS presented by Meng and Kubatko [11] and its extension to our framework. The coalescent process traces lineages backward in time until a common ancestor is reached between two lineages (a coalescent event). For large population sizes, the time to this event is well-approximated by the exponential distribution [21,22]. The coalescent model can then be applied to evolution along a species phylogeny by considering each branch of the phylogeny as an independent population [30]. This then allows calculation of probabilities of gene tree topologies given a fixed species tree with branch lengths [30,31]. Calculations carried out this way assume that ILS is the only process leading to gene tree/species tree incongruence.

Hybridization was incorporated into this setting as follows: under the assumption of a mosaic genome (where an individual gene in a hybrid organism will derive its ancestry from one of the two parental lineages) [32,33], each gene has probability *γ *of its most recent common ancestor occurring with one of the parental taxa, and probability of 1 - *γ *of its most recent common ancestor occurring with the other. As an example, Figure 1 shows a true evolutionary history for four taxa for which a hybridization event, represented by a horizontal line, has occurred. Assuming the general topology of the species tree is known, this *hybrid species tree *can be decomposed into the two species trees to the right, in which the hybrid taxon first joins one parental taxon or the other. We refer to these two trees as the *parental trees*. The model assumes that each gene traces its history through one of these two parental trees independently of all other genes. The probability of a particular gene topology given one of the two parental trees can then be assigned by the coalescent process.

Under this model, we can write the likelihood function (see also [11,13]) as follows. Given a vector, **D**, of *N *observed gene trees (e.g., **D **= (*g*_1_, *g*_2_, . . . *g_N_*)), the likelihood function for a given hybrid species tree S with the location of the putative hybridization event specified is:

(1) $$L\left(\gamma ,\;t|D,\;S\right)=\overset{N}{\underset{i=1}{\prod }}P\left(g_{i}|\gamma ,\;t,\;S\right)$$

(2) $$=\overset{N}{\underset{i=1}{\prod }}\left\{\gamma P\left(g_{i}\text{—}t,\;\tau_{1}\right)+\left(1-\gamma \right)P\left(g_{i}\text{—}t,\;\tau_{2}\right)\right\}$$

where **t **is a vector of species tree branch lengths, *τ***_1 _**and *τ***_2 _**are the two possible parental trees derived from S (see Figure 1), and *P *(*g_i _*|**t**, *τ_j_*) gives the probability of gene tree *g_i _*given parental tree *τ_j_*, which can be calculated using COAL [31]. The product is taken over genes under the assumption that the histories of the genes are independent (conditional on S and **t**). Note that the hybridization parameter, *γ*, has a direct biological interpretation as the proportion of genes in the hybrid species derived from a particular parental species. Estimation and tests concerning hybridization can thus be carried out on this parameter.

Because the likelihood depends on the species tree topology and branch lengths, these must be specified in computing the likelihood. We assume that the hybrid species tree is given but that the times of hybridization and speciation events are not known. We obtain maximum likelihood estimates (MLEs) of these parameters by successively optimizing them using Brent's method [23]. In optimizing the branch lengths, we found that it was necessary to set an upper bound on the maximum value for the branch length, since as branch lengths grow, the probability of observing a gene tree that differs from the species tree goes to 0, and the likelihood surface becomes flat. We used an upper bound of 5.0 coalescent units for the simulation study described below, and a bound of 2.0 coalescent units for the empirical study.

A likelihood ratio test can be used to examine whether there is evidence of hybridization in the presence of ILS. In particular, the likelihood ratio test statistic Δ for testing *H*_0 _: *γ *= 0 versus *H_a _*: *γ *≠ 0 is:

(3) $$\Delta =-\text{2ln}\frac{L\text{(}\gamma_{\text{0}}\text{ = 0,}{\hat{t}}_{{}_{\text{0}}}\text{—}Data\text{)}}{L\text{(}{\hat{\gamma }}_{MLE}\text{,}{\hat{t}}_{{}_{MLE}}\text{—}Data\text{)}}$$

where ${\hat{t}}_{{}_{0}}$ is the MLE of the branch length vector **t **when *γ *= 0 and ${\hat{t}}_{{}_{MLE}}$is the MLE in the unrestricted case. The test is carried out by comparing Δ to a 50:50 mixture of $\chi_{\text{1}}^{\text{2}}$ and a point mass at 0 [34].

While the overall model used in this study is similar to that proposed in Meng and Kubatko [11], we have made two important changes in our implementation. First, we have allowed for the inclusion of multiple sampled lineages within each species. Second, we have replaced the naive grid search for MLEs of the parameters with a formal optimization technique. A perl script implementing this method is available at http://www.stat.osu.edu/~lkubatko/software/HybTree/. Given a fixed species tree and an identified hybrid taxon, the perl script optimizes one parameter at a time: first *γ*, then *t*_1_, then *t*_2_, then *t*_3_. It repeats this optimization until specified convergence criteria have been reached (these may be adjusted in the perl script). The optimization procedure is that of Brent [23], a method which uses a combination of parabolic interpolation and golden section search to find extrema within a bounded interval (see Press [24] for details).

### Simulation Study

#### Data Generation

We applied this model to simulated sequence data in order to examine the ability of the method to estimate *γ *and to evaluate the power of the likelihood ratio test. One hundred trials were performed for each value of *γ *(*γ *= 0.0, 0.1, 0.3, or 0.5) for data simulated from the hybrid species tree shown in Figure 1.

For each of the 100 trials for each value of *γ*, we first determined how many gene trees were derived from each of the two parental species trees by drawing a value from a Binomial distribution with 10 trials (corresponding to a total sample of *N *= 10 loci) and probability *γ *of success. We then generated gene trees with fourteen tips given each of the parental species trees with the standard coalescent approach using Hudson's MS program [35]. The selection of fourteen tips was motivated by the data we had available to address the question of hybridization in *Sistrurus*, and the allocation to species across taxa mimics that design: there were four taxa simulated from each of the three ingroup species, and two taxa simulated from the outgroup species. In ms, we used an island model with no migration between equally-sized subpopulations following speciation. Within each subpopulation, we assumed constant population size, no recombination, and panmixis. The mutation parameter (*θ *= 4*N*_e_*μ*, where *N*_e _is the effective population size and *μ *is the neutral mutation rate) was set to a per locus value of 5.0. Branch lengths within the parental trees were set to 1.0 coalescent units (number of 2*N*_e _generations). We selected 1.0 for the branch lengths as this will result in moderate levels of ILS (see, for example, Degnan and Rosenberg [36]).

Finally, we used the gene trees obtained by ms as input for the sequence evolution program Seq-Gen [37]. Five hundred base pairs per locus were generated under the Jukes-Cantor (JC69) model with branch lengths scaled by 0.005 to convert coalescent units to mutation units.

#### Analysis of Simulated Data

We used the program PAUP* [26] to estimate gene trees from the DNA sequence data for each locus under the maximum likelihood criterion. We assumed the Jukes-Cantor model as well as the molecular clock. By assuming the same model as that used to generate the data, we remove the effect of model misspecification as a source of error in our simulation study. For the heuristic search for the ML tree, a single random addition sequence in the stepwise addition procedure was used with TBR (tree bisection-reconnection) branch swapping. In some cases, PAUP* returned more than one gene tree as the ML tree (indicating a tie in likelihood score for two or more topologies). When two or more trees were tied for the ML score, a semi-strict majority rule consensus of all tied trees was constructed and used as the estimated gene tree for that locus.

For each simulated data set, we obtained MLEs of the parameters as described above. The average and standard deviation of these parameters over the 100 trials are shown in Table 1. We also carried out a likelihood ratio test for each data set at the *α *= 0.05 level. Also shown in Table 1 is the power of this test, i.e., the percentage of trials for which the null hypothesis that *γ *= 0 was rejected.

To examine the performance of other species tree inference methods that utilize the coalescent model but do not explicitly model hybridization, we also analyzed the data with the program BEST [6]. In BEST, the prior for *θ *was set to an inverse gamma distribution with a shape parameter of 3.0 and a scale parameter of 0.003, which results in a mean of 0.0015 and a standard deviation of 0.00075. The gene mutation prior was set to a uniform distribution from 0.5 to 1.5 (the default), which allows a threefold difference in relative rates of variation across genes (see [38] for details on selection of prior distributions in BEST). Four chains were implemented within each of two runs for a maximum of 100,000,000 generations, with samples collected every 100 iterations. Convergence of the MCMC was monitored by running the analysis until the average standard deviation of split frequencies dropped below 0.03, and the first 25% of replicates were discarded as burnin. We examined the posterior probabilities of the nodes involved in the hybridization event to determine whether BEST captures information about hybridization. These results are shown in Table 3.

### Empirical Data

DNA sequence data for twelve genes (A, ATP, 1, 4, 11, 25, 31, 41, 61, 63, ETS, and GAPD - see Table 2 in [16] for details about variability within loci and [39] for information about primers used to obtain sequences) were collected for fourteen individuals - four from *S. c. catenatus *(individuals Sca 151, Sca 156, Sca 806, and Sca 88 in Table 1 of [16]), four from *S. c. tergeminus *(individuals Scter 16, Scter 02, Scter 49, Scter 83 in Table 1 in [16]), four from the putative hybrid zone (individuals Scter 30, Scter 39, Scter 44, Scter 48 which were sequenced for this study), and one individual each from *Agkistrodon contortix *(individual Agc 01 in Table 1 of [16]) and *A. piscivorus *(individual Agp 01 in Table 1 of [16]). These latter two individuals served as outgroup sequences to root the species tree. These twelve loci were chosen because they showed polymorphism within and differentiation between the putative parental taxa. The sequences from these loci in three of the four putative hybrid individuals have been deposited into Genbank (JN241640-JN241676). Accession numbers for all other sequences used in this study (including the fourth putative hybrid individual) are given in [39].

MrBayes [40] was used to obtain estimates of the gene tree for each gene under the GTR model, using the 50% majority-rule consensus of trees sampled from the posterior distribution from a run of one million generations with every 100*^th ^*tree retained after discarding the first 25% trees as burn-in. The 12 gene trees were then used by our method to estimate *γ*, as well as speciation times and the time of the hybridization event, as described above. The empirical data were also analyzed using the program BEST, with the same settings as used in the simulation study described above.

## Authors' contributions

DG wrote the perl scripts to carry out the analysis, carried out the simulation study, analyzed the empirical data, and wrote the first draft of the manuscript. HLG and LK supervised the research project, and assisted with writing the manuscript. All authors have read and approved the final manuscript.

## References

[B1] Mallet J Hybrid speciation Nature 2007 446 279 283 10.1038/nature05706 17361174

[B2] Hobolth A Christensen OF Schierup MH Genomic relationships and speciation times of human, chimpanzee, and gorilla inferred from a coalescent hidden Markov model PLoS genetics 2007 3 294 304 10.1371/journal.pgen.0030007 PMC1802818 17319744

[B3] Than C Identifiability issues in phylogeny-based detection of horizontal gene transfer Comparative Genomics, Proceedings 2006 4205 215 229 10.1007/11864127_17

[B4] Guigo R Muchnik I Smith TF Reconstruction of ancient molecular phylogeny Molecular Phylogenetics and Evolution 1996 6 189 213 10.1006/mpev.1996.0071 8899723

[B5] Edwards SV Is a new and general theory of molecular systematics emerging? Evolution 2009 63 1 19 10.1111/j.1558-5646.2008.00549.x 19146594

[B6] Liu L Pearl D Species trees from gene trees: reconstructing Bayesian posterior distributions of a species phylogeny using estimated gene tree distributions Syst Biol 2007 56 504 514 10.1080/10635150701429982 17562474

[B7] Heled J Drummond AJ Bayesian inference of species trees from multilocus data Mol Biol Evol 2010 27 3 570 580 10.1093/molbev/msp274 19906793 PMC2822290

[B8] Than C Nakhleh L Species tree inference by minimizing deep coalescences PLoS Comp Biol 2009 5 9 e1000501. 10.1371/journal.pcbi.1000501 PMC2729383 19749978

[B9] Kubatko L Carstens B Knowles L STEM: Species tree estimation using Maximum likelihood for gene trees under coalescence Bioinformatics 2009 25 971 97 10.1093/bioinformatics/btp079 19211573

[B10] Buckley T Cordeiro M Marshall D Simon C Differentiating between hypotheses of lineage sorting and introgression in New Zealand cicadas (Maoricicada dugdale) Syst Biol 2006 55 411 425 10.1080/10635150600697283 16684720

[B11] Meng C Kubatko LS Detecting hybrid speciation in the presence of incomplete lineage sorting using gene tree incongruence: A model Theor Pop Biol 2009 75 35 45 10.1016/j.tpb.2008.10.004 19038278

[B12] Kubatko LS Identifying hybridization events in the presence of coalescence via model selection Syst Biol 2009 58 478 488 10.1093/sysbio/syp055 20525602

[B13] Nakhleh L Heath L, Ramakrishnan N Evolutionary phylogenetic networks: models and issues The Problem Solving Handbook for Computational Biology and Bioinformatics 2010 Springer 125 158

[B14] Joly S McLenachan PA Lockhart PJ A statistical approach for distinguishing hybridization and incomplete lineage sorting American Naturalist 2009 174 2 E54 70 10.1086/600082 19519219

[B15] Yu Y Than C Degnan J Nakhleh L Coalescent histories on phylogenetic networks and detection of hybridization despite incomplete lineage sorting Systematic Biology 2011 60 138 149 10.1093/sysbio/syq084 21248369 PMC3167682

[B16] Kubatko LS Gibbs HL Bloomquist EW Inferring species-level phylogenies and taxonomic distinctiveness using multi-locus data in Sistrurus rattlesnakes Syst. Biol 2011 60 393 409 10.1093/sysbio/syr011 21389297

[B17] Gloyd HK The Rattlesnakes, Genera Sistrurus And Crotalus Special Publications of the Chicago Academy of Sciences 1940 4 104 118

[B18] Evans PD Gloyd HK The subspecies of the massasauga, *Sistrurus catenatus, in Missouri* Bulletin of the chicago academy of sciences 1948 8 225 232

[B19] Gibbs HL Murphy M Chiucchi JE Genetic identity of endangered massasauga rattlesnakes (Sistrurus sp.) in Missouri Conservation Genetics 2010 12 2 433 439

[B20] Szymanski J Status assessment for the Eastern *Massasauga (Sistrurus c. catenatus) 1998* U.S. Fish and Wildlife Service, Endangered Species Division, Fort Snelling, MN 1998

[B21] Kingman JFC On the genealogy of large populations J Appl Prob 1982 19A 27 43

[B22] Kingman JFC The coalescent Stoch Proc Appl 1982 13 235 248 10.1016/0304-4149(82)90011-4

[B23] Brent RP Algorithms for Minimization Without Derivatives 1973 Prentice-Hall

[B24] Press WH Numerical recipes in fortran 77: the art of scientific computing 1986 1 Cambridge University Press

[B25] Rosenberg NA The shapes of neutral gene genealogies in two species: probabilities of monophyly, paraphyly, and polyphyly in a coalescent model Evolution 2003 57 7 1465 1477 12940352 10.1111/j.0014-3820.2003.tb00355.x

[B26] Swofford DL PAUP*. Phylogenetic Analysis Using Parsimony (*and Other Methods). Version 4 2003

[B27] Felsenstein J PHYLIP (Phylogeny Inference Package) version 3.6 Distributed by the author Department of Genome Sciences, University of Washington, Seattle 2005

[B28] Fan H Kubatko LS Estimating species trees using approximate Bayesian computation Mol Phylogen Evol 2011 59 354 363 10.1016/j.ympev.2011.02.019 21397706

[B29] Kubatko LS Meng C Knowles LL, Kubatko LS Accomodating hybridization in a multilocus phylogenetic framework Estimating Species Trees: Practical and Theoretical Aspects 2010 Wiley-Blackwell, New Jersey 99 113

[B30] Pamilo P Nei M Relationships between gene trees and species trees Mol Biol Evol 1988 5 5 568 583 3193878 10.1093/oxfordjournals.molbev.a040517

[B31] Degnan J Salter L Gene tree distributions under the coalescent process Evolution 2005 59 24 37 15792224

[B32] Rieseberg L Wendel J RG Harrison Introgression and its consequences in plants Hybrid Zones and the Evolutionary Process 1993 Oxford University Press 70 109

[B33] Rieseberg L Hybrid origins of plant species Annu Rev Ecol Syst 1997 28 359 389 10.1146/annurev.ecolsys.28.1.359

[B34] Self S Liang KY Asymptotic properties of maximum likelihood estimators and likelihood ratio tests under nonstandard conditions J Am Stat Assoc 1987 82 605 610 10.2307/2289471

[B35] Hudson RR Generating samples under a Wright-Fisher neutral model Bioinformatics 2002 337 338 10.1093/bioinformatics/18.2.337 11847089

[B36] Degnan JH Rosenberg NA Discordance of species trees with their most likely gene trees PLoS Genetics 2006 3 762 768 10.1371/journal.pgen.0020068 PMC1464820 16733550

[B37] Rambaut A Grassly N SeqGen: An application for the Monte Carlo simulation of DNA sequence evolution along phylogenetic trees Comput Appl in Biosci 1997 13 235 238 10.1093/bioinformatics/13.3.235 9183526

[B38] Castillo-Ramirez S Liu L Pearl D Edwards SV Knowles LL, Kubatko LS Bayesian estimation of species trees: a practical guide to optimal sampling and analysis Estimating Species Trees: Practical and Theoretical Aspects 2010 Wiley-Blackwell, New Jersey 15 33

[B39] Gibbs HL Diaz J Identification of single copy nuclear DNA markers for North American pit vipers Molecular Ecology Resources 2010 10 177 180 10.1111/j.1755-0998.2009.02707.x 21565006

[B40] Ronquist F Huelsenbeck JP MrBayes3: Bayesian phylogenetic inference under mixed models Bioinformatics 2003 19 1572 1574 10.1093/bioinformatics/btg180 12912839

